# EZH2-dependent epigenetic modulation of histone H3 lysine-27 contributes to psoriasis by promoting keratinocyte proliferation

**DOI:** 10.1038/s41419-020-03028-1

**Published:** 2020-10-03

**Authors:** Tongmei Zhang, Luting Yang, Yao Ke, Jie Lei, Shengxian Shen, Shuai Shao, Chen Zhang, Zhenlai Zhu, Erle Dang, Gang Wang

**Affiliations:** Department of Dermatology, Xijing Hospital, Fourth Military Medical University, Xi’an, China

**Keywords:** Psoriasis, Chronic inflammation

## Abstract

Psoriasis is characterized by keratinocyte hyperproliferation. While significant progress has been made in understanding the molecular mechanism regulating the proliferation of keratinocytes, little is known about the epigenetic factors that control this process. EZH2 and EZH2 mediated trimethylation of histone H3 lysine 27 (H3K27me3) was previously shown ectopically expressed in carcinoma and mediated proliferation, thereby we sought to clarify the role of EZH2–H3K27me3 in the proliferation of psoriatic keratinocyte. Interestingly, we found that EZH2 and H3K27me3 were both overexpressed in the epidermis of psoriatic lesional skin compared to normal skin. In vitro, the expression of EZH2 and H3K27me3 was stimulated in human keratinocytes treated with mixture of psoriasis-related cytokines pool (TNF-α, IFN-γ, IL-17A, and IL-22). Knockdown of EZH2 significantly reduced keratinocyte proliferative activity. Results from mRNA microarray analysis suggested that Kallikrein-8 (KLK8) might be the target gene of EZH2 in psoriatic keratinocytes. Overexpression or knockdown KLK8 could partially reverse the abnormal proliferation of keratinocytes caused by knockdown or overexpression of EZH2. In vivo, the inhibitor of EZH2, GSK126 could ameliorate the imiquimod-induced psoriasiform lesion. These results suggest that EZH2 might be a therapeutic target for the treatment of psoriasis.

## Introduction

Psoriasis is a common chronic immune dermatosis characterized by hyperproliferation of keratinocytes. Despite increasing understanding of the pathogenesis of psoriasis, the regulation mechanism relating to its abnormal proliferation has not yet been fully elucidated. Recent studies have demonstrated that proinflammatory cytokines, such as IL-22^1,2^, IL-17^3,4^, IL-1^5^, and IGF^6^ secreted by some autocrine or local inflammatory environment could activate cell proliferation-related signaling pathways, which accelerated cell proliferation and delayed differentiation, leading to epidermal hyperplasia in psoriasis^7,8^.

The case of identical twins with one suffering from psoriasis while the other not suggests that epigenetic regulation might be implicated in the pathogenesis of psoriasis^9^. Epigenetic regulation mainly includes DNA methylation, histone modification, and miRNAs regulation. As an essential epigenetic regulation, histone methylation plays key roles in the regulation of gene expression, embryo development, and genome reprogramming. Abnormalities in histone methylation could trigger tumorigenesis, such as breast cancer, prostate cancer, lung cancer, and glioma.

Histone methylation modification occurs mainly on lysine (Lys, K) and arginine (Arg, R) residues. In general, methylation of H3K4, H3K36, H3K79, and H3R17 are associated with activation of gene expression, while methylation of H3K9, H3K27, and H4K20 sites are associated with gene silencing^10–13^. Histone methylases and demethylases could reversibly alter histone methylation status and affect transcriptional activation or silencing of the target gene. Enhancer of zeste homolog 1 (EZH1) and 2 (EZH2) are both histone H3K27 methylases that catalyzes the trimethylation of H3K27^14,15^. EZH2, which affected the global level of H3K27me3, is implicated in cell proliferation and tumor growth^15,16^. Studies have shown that by silencing specific genes, H3K27me3 could promote tumor development^17,18^. EZH2 and H3K27me3 levels were significantly elevated in poorly differentiated head and neck squamous cell carcinoma (HNSCC), and inhibition of EZH2 could induce differentiation-related gene expression^16^. In the study of epidermal cells, depletion of EZH2 could reduce the level of H3K27me3 in epidermal cells, accompanying with reduced cell proliferation and survival, leading to premature differentiation of epidermis^19^. These results suggested that EZH2 catalyzed H3K27me3 might affect epidermal proliferation. Considering that psoriasis is an inflammatory skin disease characterized also by epidermal hyperproliferation, it is hypothesized that EZH2 might affect the keratinocyte proliferation in psoriasis through epigenetic modification. In this report, we showed that EZH2 promoted the proliferation of keratinocyte through KLK8, and inhibition of EZH2 had a therapeutic effect on psoriasis in imiquimod-induced psoriasis-like mouse model. Our research provides a theoretical basis for the treatment of psoriasis by targeting EZH2.

## Results

### EZH2 and H3K27me3 were both upregulated in psoriasis epidermis and keratinocytes stimulated by psoriasis-related mixed cytokines in vitro

**Fig. 1:**
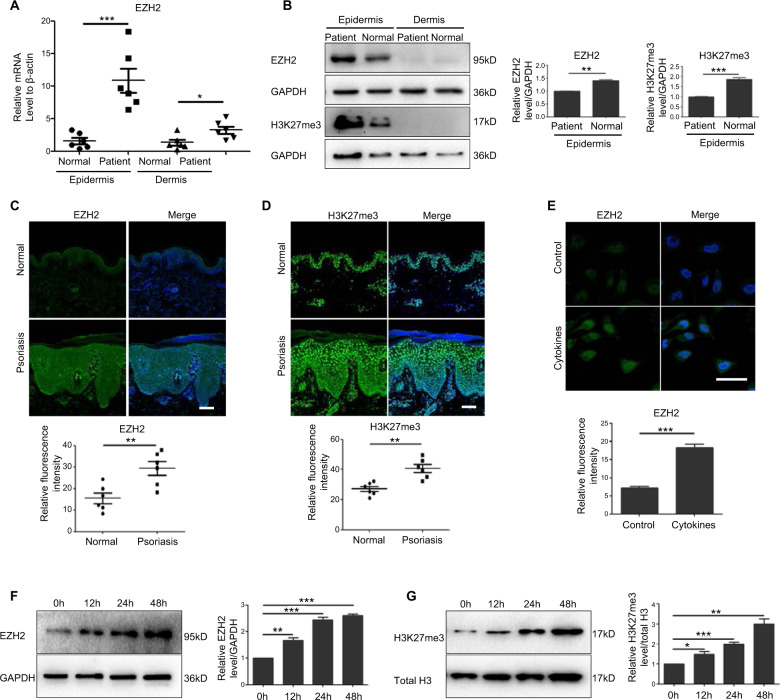
EZH2 and H3K27me3 were both upregulated in psoriasis epidermis. Epidermis and dermis from six psoriasis lesional skin and six healthy controls were separated. **a** EZH2 mRNA levels were detected by Real-Time PCR. **b** Protein levels of EZH2 and H3K27me3 were detected by Western Blotting. Band intensities were quantified relative to GAPDH. **c**, **d** Immunofluorescent analysis of EZH2 and H3K27me3 compared with normal skin. Scale bar = 100 μm. **e** Immunofluorescent analysis of EZH2 in HaCat cells after treatment of psoriasis-related mixed cytokines (TNF-α 50 ng/ml, IFN-γ 20 ng/ml, IL-17A 30 ng/ml, and IL-22 30 ng/ml) for 24 h. Scale bar = 50 μm. **f**, **g** HaCat cells were treated with mixed cytokines for 12, 24, and 48 h. EZH2 and H3K27me3 levels were detected by Western Blotting. Band intensities were quantified relative to GAPDH or total H3. *P < 0.05; **P < 0.01; ***P < 0.001.

Expression of EZH2 and H3K27me3 were compared in samples from six psoriasis lesional skin and six healthy controls by Real-Time PCR (Fig. 1a), western blotting (Fig. 1b) and immunofluorescence staining (Fig. 1c, d).

The results showed that the expression of EZH2 and H3K27me3 were significantly upregulated in the epidermis of psoriasis lesional skin, whereas their expression was very low in dermis of skin tissues and no difference in protein levels was discovered between psoriasis patients and normal. Consistent with western blotting analysis, immunofluorescence signals showed a 2.98 ± 0.46 fold increase of EZH2 and 1.74 ± 0.12 fold increase of H3K27me3, respectively in psoriatic epidermis. In summary, these results suggested that both the expression of EZH2 and H3K27me3 were upregulated in the epidermis of psoriatic patients. To investigate the impact of EZH2 on keratinocyte proliferation, we employed HaCaT cells, an immortalized human keratinocytes cell line. Previous reports showed that stimulation of HaCaT cells by psoriasis-related mixed cytokines (TNF-α, IFN-γ, IL-17A, and IL-22) could mimic in vitro the psoriasis-like condition^20^, we thus conducted our experiments in this model. Results from immunofluorescence showed that the expression of EZH2 in HaCaT cells was significantly upregulated after treatment with psoriasis-related mixed cytokines for 24 h (Fig. 1e). To further examine whether the upregulation induced by mixed cytokines was time-dependent, we performed a time-course stimulation with the aforementioned cytokines. The results showed that the expression of EZH2 and H3K27me3 gradually increased with time, with a maximal protein level observed at 48 h poststimulation (Fig. 1f, g).

### EZH2 regulated proliferation of keratinocytes

**Fig. 2:**
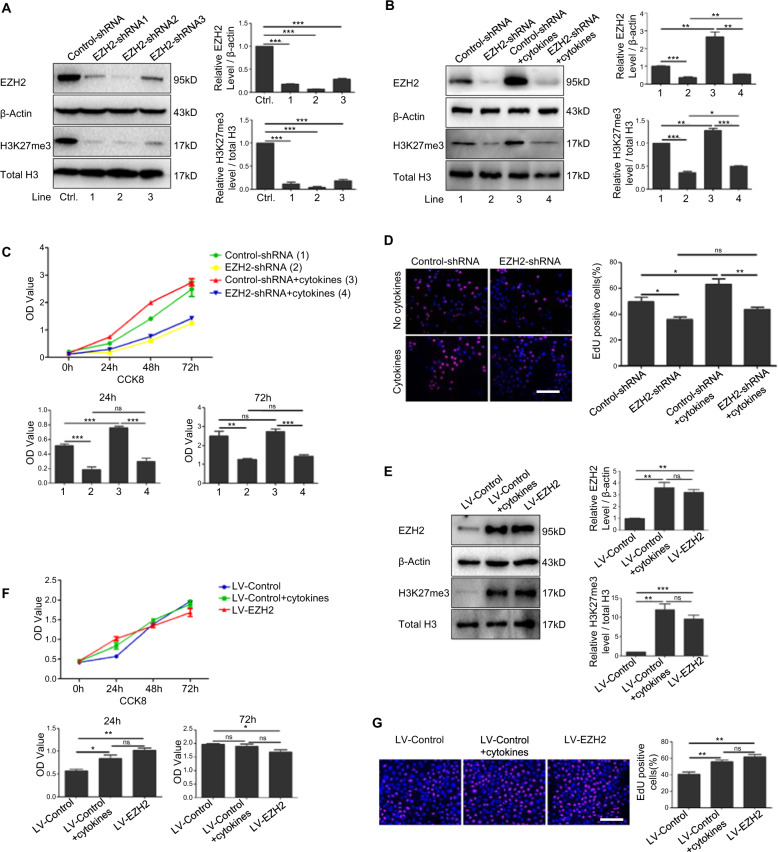
EZH2 regulated proliferation of keratinocytes. **a** HaCat cells were infected with lentiviral non-targeting control (Control-shRNA) or ShRNA targeting EZH2 (EZH2-shRNA1, 2, and 3). The efficiency of EZH2 and H3K27me3 knockdown was verified by Western Blotting. **b** HaCat cells infected with Control-shRNA or EZH2–shRNA2 were treated with mixed cytokines. EZH2 and H3K27me3 protein levels were detected by Western Blotting. Band intensities were quantified relative to β-actin or total H3. Proliferation of cells was analyzed by Cell Counting Kit-8 (**c**) and EdU assay (**d**). Scale bar = 100 μm. **e** Cells were subjected to lentiviral infection to overexpress EZH2. The effect of overexpressing EZH2 and H3K27me3 was detected by Western Blotting. Band intensities were quantified relative to β-actin or total H3. Proliferation of cells was analyzed by Cell Counting Kit-8 (**f**) and EdU assay (**g**). Scale bar = 100 μm. *P < 0.05; **P < 0.01; ***P < 0.001.

To further clarify the effect of EZH2 on keratinocyte proliferation, we employed lentivirus to downregulate or overexpress the expression of EZH2 in HaCaT cells. Firstly, the efficiency of EZH2 knockdown was confirmed by western blotting after infection with EZH2 shRNA1, 2, and 3. As EZH2–shRNA2 (EZH2–shRNA) reached the highest effect for the reduction of EZH2, it was selected for subsequent experiments (Fig. 2a). Furthermore, in HaCaT cells and psoriatic HaCaT cells which is stimulated with psoriasis-related mixed cytokines, knockdown of EZH2 could significantly reduce the expression of H3K27me3 (Fig. 2b). We further analyze whether EZH2 had a potential role in regulating the proliferation of keratinocytes. CCK8 analysis (Fig. 2c) and EdU assays (Fig. 2d) showed that the proliferation of HaCaT cells was significantly reduced after knockdown of EZH2, regardless of whether these cells are stimulated with psoriasis-related mixed cytokines. Consistently, overexpression of EZH2 increased the expression of H3K27me3 and the proliferation of HaCaT cells (Fig. 2e–g). All these results implied a favorable role of EZH2 in promoting the proliferation of keratinocytes.

### KLK8 was identified as a downstream gene of EZH2 in keratinocytes

**Fig. 3:**
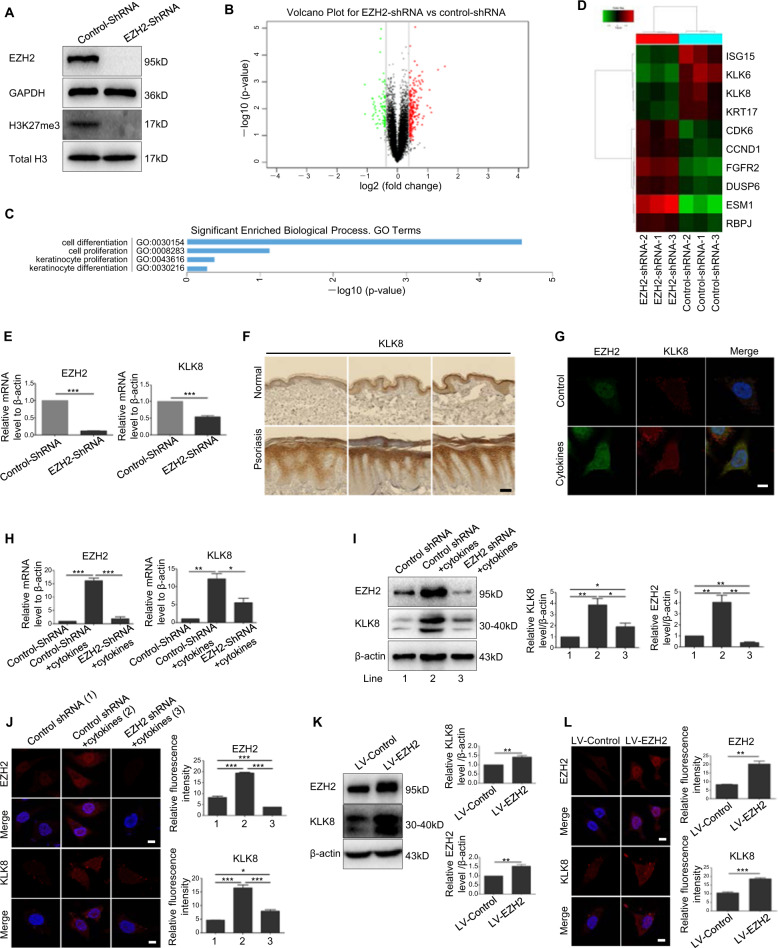
KLK8 was identified as a downstream gene of EZH2 in keratinocytes. **a** EZH2–shRNA2 (EZH2–shRNA) was chosen to construct the stable EZH2 knockdown HaCat cell line by puromycin. EZH2 was totally knocked out in these cells. **b** mRNA microarray analysis identified 344 genes closely related to EZH2. **c** EZH2 was involved in cell proliferation and differentiation. **d** Gene thermal map of ten selected genes. **e** KLK8 and EZH2 mRNA levels were detected in the original samples using Real-Time PCR. **f** Immunohistochemical staining of KLK8 in psoriatic lesional skin and normal skin. Scale bar = 100 μm. **g** Colocalization of KLK8 with EZH2 by immunofluroescence in HaCaT cells treated with mixed cytokines. Scale bar = 10 μm. HaCaT cells were infected with EZH2–shRNA or Control-shRNA and treated with mixed cytokines. mRNA and protein levels of KLK8 and EZH2 were detected by Real-Time PCR (**h**) or Western Blotting (**i**), respectively. **j** Immunofluorescence analysis of KLK8, EZH2 after knockdown of EZH2 and treatment of mixed cytokines. Scale bar = 10 μm. **k** After HaCaT cells were infected with lentiviras overexpressing EZH2 (LV-EZH2) or control lentiviras (LV-Control), KLK8 and EZH2 protein levels were detected. **l** Immunofluorescence analysis of KLK8, EZH2 after overexpression of EZH2. Scale bar = 10 μm. *P < 0.05; **P < 0.01; ***P < 0.001.

To explore the molecular mechanism by which EZH2 modulated keratinocyte proliferation, we employed microarray analysis. Differentially expressed genes were analyzed in EZH2–shRNA and control HaCaT cells (Fig. 3a). From the data of microarray analysis, 344 differentially expressed genes were found between EZH2–shRNA and control group using 1.3-fold cutoff. Of these genes, 254 genes were upregulated in EZH2–shRNA group and 90 genes were down-regulated (Fig. 3b). Gene ontology analysis suggested that silencing of EZH2 mainly affected the proliferation and differentiation of keratinocytes (Fig. 3c). We screened ten genes associated with proliferation and differentiation, and mapped them into gene thermal map (Fig. 3d). Real-Time PCR was employed to detect these ten genes in original samples. Among these genes, KLK8, a member of kallikrein-related peptidases implicated in cell proliferation and proteolytic cascade in the skin^21^, decreased at the most obvious level after knockdown of EZH2 (Fig. 3e and Supplementary Fig. 1). Then we detected the expression of KLK8 from skin tissue sample of psoriasis patients and healthy controls. Results from immunohistochemical staining showed that the expression of KLK8 was increased greatly in epidermis of psoriasis lesional skin, which is consistent with the change of EZH2 (Fig. 3f). Moreover, KLK8 could also be stimulated by psoriasis-related mixed cytokines (Fig. 3g). In order to investigate whether KLK8 is regulated by EZH2 in a psoriatic-like microenvironment, cells were infected with EZH2–shRNA following stimulation with mixed cytokines. Consistently, in psoriatic keratinocytes, knockdown of EZH2 induced a decrease in KLK8 (Fig. 3h, i). Moreover, immunofluorescence showed a reduced fluorescent intensity of KLK8 after knockdown of EZH2 in HaCaT cells stimulated with mixed cytokines (Fig. 3j). On the opposite, overexpression of EZH2 increased the expression of KLK8 (Fig. 3k, l). The above results suggested that KLK8 might a potential downstream target of EZH2 in psoriatic keratinocytes.

### KLK8 regulated proliferation of keratinocytes

**Fig. 4:**
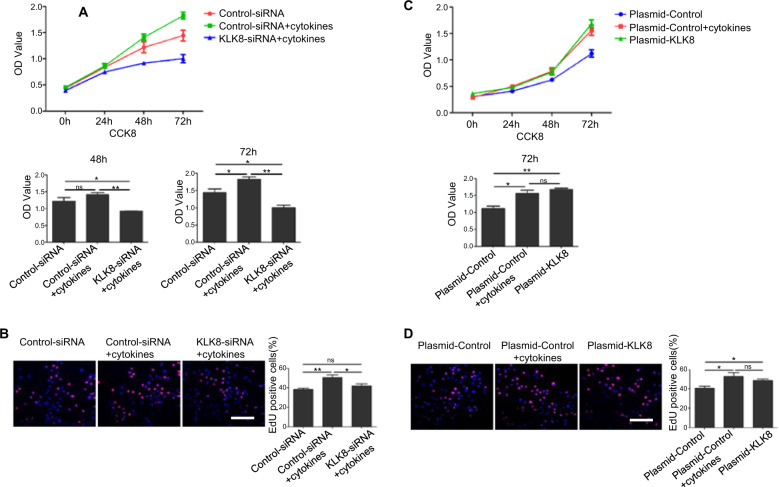
KLK8 regulated proliferation of keratinocytes. HaCat cells were transfected with siRNA for KLK8 (KLK8-siRNA) or control siRNA, and treated with mixed cytokines. Proliferation of cells was analyzed by Cell Counting Kit-8 (**a**) and EdU assay (**b**). Scale bar = 100 μm. Plasmid for overexpression of KLK8 (Plasmid-KLK8) or control plasmid (Plasmid-Control) was transfected in HaCaT cells. Proliferation of cells was analyzed by Cell Counting Kit-8 (**c**) and EdU assay (**d**). Scale bar = 100 μm. *P < 0.05; **P < 0.01.

We further explored the effect of KLK8 on the proliferation of keratinocytes. siRNA specific to KLK8 was used to knock down the expression of KLK8 in HaCaT cells stimulated with mixed cytokines (Supplementary Fig. 2A). CCK8 analysis (Fig. 4a) and EdU assays (Fig. 4b) showed knockdown of KLK8 significantly reduced the proliferation of psoriatic HaCaT cells. Furthermore, overexpression of KLK8 in HaCaT cells significantly promoted cells proliferation compared with the normal cells, the effect was equivalent to cytokines stimulation group (Supplementary Fig. 2B and Fig. 4c, d). These results suggested that KLK8 could promote the proliferation of keratinocytes.

### EZH2 might promote keratinocytes proliferation through upregulating the expression of KLK8

**Fig. 5:**
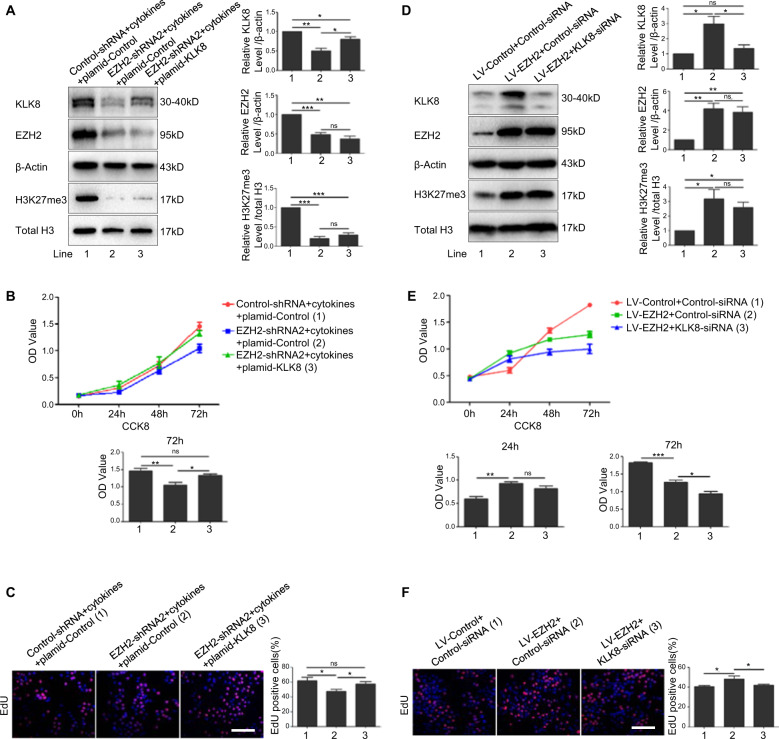
EZH2 promote keratinocytes proliferation through upregulating the expression of KLK8. Plasmid-KLK8 or Plasmid-Control was transfected in HaCaT cells which were infected with EZH2–shRNA and treated with mixed cytokines. **a** Protein levels of KLK8, EZH2, and H3K27me3 were detected by Western Blotting. Proliferation of cells was analyzed by Cell Counting Kit-8 (**b**) and EdU assay (**c**). Scale bar = 100 μm. KLK8-siRNA or control-siRNA was transfected in EZH2-overexpressing HaCaT cells. **d** Protein levels of KLK8, EZH2 and H3K27me3 were detected by Western Blotting. Proliferation of cells was analyzed by Cell Counting Kit-8 (**e**) and EdU assay (**f**). Scale bar = 100 μm. *P < 0.05; **P < 0.01; ***P < 0.001.

To investigate whether EZH2 affects cell proliferation via upregulation of KLK8 in a psoriatic-like microenvironment, we overexpressed the expression of KLK8 in EZH2 knockdown HaCaT cells stimulated with mixed cytokines (Fig. 5a). Results from CCK8 analysis (Fig. 5b) and EdU assays (Fig. 5c) showed that overexpression of KLK8 partially rescued the reduced proliferation of HaCaT cells induced by knockdown of EZH2 in a psoriatic-like microenvironment. Furthermore, KLK8 was knocked down in cell overexpressed with EZH2 (Fig. 5d). Results from CCK8 analysis (Fig. 5e) and EdU assays (Fig. 5f) showed that knockdown of KLK8 partially rescued the increased proliferation of HaCaT cells caused by overexpression of EZH2. All these results showed that overexpression or knockdown KLK8 could partially reverse the abnormal proliferation of keratinocytes caused by knockdown or overexpression of EZH2, which indicated that EZH2 might signal through upregulating the expression of KLK8 to promote the proliferation of keratinocytes. However, whether KLK8 is a direct downstream target of EZH2 are need to be further investigated.

### GSK126, inhibitor of EZH2, attenuated the phenotype of imiquimod-induced psoriasis-like mouse model

**Fig. 6:**
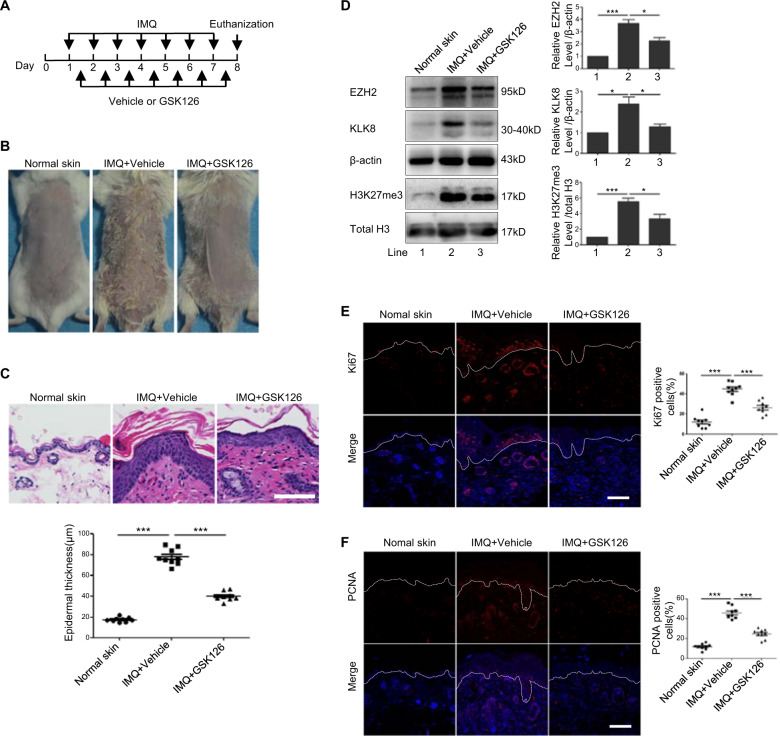
EZH2 inhibitor improved psoriatic phenotype induced by IMQ in mice. **a** Mice were topically treated daily with IMQ at 8:00 am and with GSK126 or Vehicle respectively at 16:00 pm for seven days. **b** Phenotype of mice in different groups on day 8. **c** The epidermis thickness of the back lesions of mice was observed by H&E staining (*n* = 9). **d** The protein levels of EZH2, H2K27me3, and KLK8 were detected by Western Blotting. Scale bar = 100 μm. **e**, **f** Ki67 and PCNA positive cells in the back lesions of mice were examined by Immunofluorescence analysis (*n* = 9). Scale bar = 100 μm. *P < 0.05; ***P < 0.001.

To study the effect of using EZH2 as a target for the treatment of psoriasis, we adopted GSK126, a small molecule chemical inhibitor of EZH2 to treat imiquimod-induced psoriasis-like mouse model. We firstly verified in vitro that application of GSK126 could significantly inhibit the elevation of H3K27me3 caused by mixed cytokines stimulation, and weaken the proliferation activated by mixed cytokines (Supplementary fig. 3). The experimental plan was shown in Fig. 6a. Seven days after the treatment, erythema and scales appeared in the imiquimod treatment group, whereas these phenotypes induced by imiquimod was ameliorated after treatment with GSK126 (Fig. 6b). H&E staining showed a markedly increased epidermal thickness after treatment with imiquimod, confirming the success of psoriasis-like mouse model induced by imiquimod (Fig. 6c). However, this increase of epidermal thickness was inhibited after treatment with GSK126 (Fig. 6c). Results from western blotting from mouse epidermis showed that the expression of EZH2, H3K27me3, and KLK8 were upregulated in the imiquimod treatment group; however, their expression was decreased after GSK126 treatment (Fig. 6d). Moreover, the proliferation of keratinocytes, represented by the staining of Ki67 and PCNA in the epidermis was significantly reduced after treatment with GSK126 (Fig. 6e, f). All these results suggested that inhibition of EZH2 attenuated the phenotype of imiquimod-induced psoriasis-like mouse model.

## Discussion

Epigenetic regulation plays an important role in the pathogenesis of psoriasis. Recent years, a large number of studies have focused on DNA methylation. Abnormal DNA methylation in the epidermis, dermis and Peripheral blood mononuclear cells (PBMC) of psoriasis is closely related to the pathogenesis of psoriasis. DNA methylation on CpG island causes the reduction of the protein SFRP4, which in turn increases the Wnt pathway, causing the proliferation of keratinocytes in psoriasis^22^. Epidermal DNA methylation returns to normal after NB-UVB phototherapy in patients with psoriasis^23^. On the basis of abnormal DNA methylation of psoriasis PBMC^24^, another study found that the DNA methylation level of T lymphocytes in PBMC also increased^25^.

Histone modifications including acetylation, methylation, ubiquitination, and phosphorylation also play an important role in the pathogenesis of psoriasis. One study showed that H4 in psoriasis PBMC was hypoacetylated^26^, which was negatively correlated with PASI score of psoriasis^27^. The role of histone methylation in psoriasis has also been reported. Chen et al. found that Grainyhead-like 2 (GRHL2), a novel transcription factor, inhibits keratinocytes differentiation by inhibiting genes in the epidermal differentiation complex (EDC)^28^. The abnormal expression of GRHL2 in the psoriasis epidermis prevents the recruitment of demethylase JMJD3 to the EDC gene promoters and enhances the H3K27me3 level in gene promoter, thus causing cell hyperproliferation^28^. In the primary T lymphocytes of psoriasis patients who were positive for the psoriasis susceptibility gene site *PSORS1*, three gene loci within *PSORS1* had been detected with H3K4me1 and H3K27ac markers^29^. All these studies suggest that histone methylation modification plays an important role in the pathogenesis of psoriasis.

In our research, we focused on the role of EZH2 in catalyzing H3K27me3 in keratinocytes of psoriasis. EZH2, an important component of the PRC2 complex, can catalyze the trimethylation of H3K27. And EZH1, as a homolog of EZH2, can form a similar PRC2 complex and also has an effect on the methylation of H3K27. They both lead to transcriptional inhibition of the target genes, but their efficiency is different. Knockdown of EZH2 affects global H3K27me3 level, while knockdown of EZH1 has no effect on global H3K27me3 level^15^. EZH1 is detected in nonproliferative adult organs, while the expression of Ezh2 is closely associated with cell proliferation^15^. At the beginning of our study, we have detected the level of EZH1 mRNA in psoriasis lesional skin. Results showed that there was no significant difference in EZH1 between psoriatic lesions and normal skin (Supplementary Fig. 4). So we did not continue to explore EZH1 in the follow-up research.

In our study, we found that EZH2 and H3K27me3 were significantly increased in psoriatic lesions, especially in the epidermis. However, our team showed that in CD3 T lymphocytes from PBMC of psoriatic patients was significantly lower than that of normal people and negatively correlated with PASI score (Supplementary Fig. 5). This finding is consistent with Ovejero-Benito M C team’s research results. They examined changes in the level of histone modification in PBMC of psoriasis patients before and after treatment with biological agents. They found that although H3K27 methylation in PBMC of patients was not significantly different from healthy subjects before treatment, H3K27 levels were significantly elevated in biologic responders after 3 months of treatment compared to nonresponders^30^. In another study, researchers found that EZH2 was significantly increased in psoriasis, but there was no significant difference in H3K27me3^26^. We speculate the differential results of H3K27me3 observed in PBMCs and lesional skins of psoriatic patients might be due to the post-translational and epigenetic modification of EZH2, so that the level of H3K27me3 is inconsistent with the expression of EZH2. This suggests the complexity of epigenetic regulation in psoriasis, and its function may be different in different cell subsets. In this article, we focused on the functions of H3K27me3 and EZH2 in keratinocytes. Our results showed that EZH2 could promote KC proliferation in the inflammatory environment of psoriasis in vitro.

KLK8 is a member of kallikrein-related peptidases. It has been reported that KLK8 is elevated in PsA synovial fluid and psoriatic plaque, and positively correlated with PASI score^31^. KLK8 is also involved in the formation of microabscess in imiquimod-induced psoriasis-like mice^32^. Kishibe et al. used 12-O-tetradecyl phorbol-13-acetate on the skin of *Klk8*^−/−^ mice to induce a psoriasis-like model. They found that *Klk8*^−/−^ mice had reduced epidermal proliferation compared to wild type^33^. These studies indicate that KLK8 is involved in the proliferation of keratinocytes. Our study further clarified that KLK8 can indeed promote KC proliferation under psoriatic inflammatory environment simulated in vitro, and KLK8 is regulated by EZH2. Overexpression or knockdown of KLK8 could reverse the abnormal proliferation of keratinocytes induced by knockdown or overexpression of EZH2. However, whether EZH2 interacts directly or indirectly with KLK8 to affect its expression are not clarified in this study. The link between EZH2–H3K27–KLK8 needs to be further verified in future research.

GSK126 is a highly selective, S-adenosylmethionine-competitive, small molecule inhibitor of EZH2. It can effectively reduce the level of H3K27me3^34^. GSK126 has been used in the study of lymphoma and other solid tumors to achieve the purpose of treating tumors by inhibiting EZH2. In these studies, mice were given intraperitoneal injections^34,35^. In our study, we performed the GSK topical administration for the first time. We found that topical application of GSK126 can alleviate the phenotype of imiquimod-induced psoriasis-like mouse models and reduce the rate of epidermal cell proliferation.

**Fig. 7:**
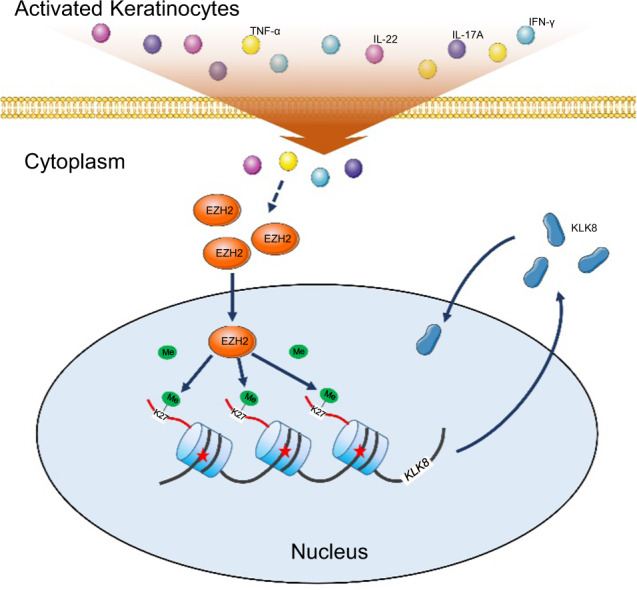
EZH2 contributes to psoriasis by promoting keratinocyte proliferation through KLK8. EZH2 is upregulated in keratinocytes in the inflammatory microenvironment of psoriasis, which catalyzes H3K27me3, and further promotes keratinocyte proliferation through upregulation of KLK8.

In conclusion, our study showed upregulated EZH2 in psoriatic epidermis, which might catalyze trimethylation of H3K27, inducing the expression of KLK8 and further promotes keratinocyte proliferation in psoriasis (Fig. 7). This study uncovers the role of EZH2-dependent epigenetic modification in the regulation of psoriatic keratinocyte hyperproliferation and clarifies the related mechanisms, providing new evidence for the involvement of histone methylation in the pathogenesis of psoriasis. EZH2 is a potential molecular target for the treatment of psoriasis and are worth attention in the future drug discovery for psoriasis.

## Materials and methods

### Cell culture

The cell line HaCat purchased from KeyGEN Biotech (Jiangsu, China) was maintained in RPMI1640 with 10% FBS. The cell was cultured with 5% CO_2_ at 37 °C. When HaCat cells grew to 30–40%, cytokines (50 ng/ml TNF-α, 20 ng/ml IFN-γ, 30 ng/ml IL-17, and 30 ng/ml IL-22) were added to the culture solution to simulate the inflammatory environment of psoriasis in vitro. GSK126 dissolved in DMSO was used at the concentration of 50 nM. The HaCat cell line was authenticated by STR profiling and tested for mycoplasma contamination.

### Antibodies and reagents

The primary antibodies used for Western blots were anti-EZH2 (D2C9, 5246S, Cell Signaling Technology, Danvers, MA), anti-H3K27me3 (Lys27) (C36B11, 9733, Cell Signaling Technology), anti-total H3 (D1H2, 4499S, Cell Signaling Technology), anti-KLK8 (14232-1-AP, Proteintech, Chicago, Illinois). Antibodies used for Immunofluorescence staining were anti-EZH2 (MA5-15101, Thermo Scientific, Waltham, MA), anti-Ki67 (8D5, 9449, Cell Signaling Technology), anti-PCNA (PC10, 2586, Cell Signaling Technology). GSK126 (SC0060) was purchased from Beyotime biotechnology (Shanghai, China). Recombinant Human IL-17 (IL-17A), IL-22, TNF-α, and IFN-γ were purchased from Peprotech (Rocky Hill, New Jersey).

### Specimens of patients and controls

After the volunteers had signed the informed consent form, their skin tissue samples were obtained through surgical procedures. Inpatients of psoriasis were selected for typical plaque lesions during the progression period and did not undergo systematic treatment at least 1 month before the operation. Patients statistics were showed in Supplementary Table 1. A typical skin lesion on the trunk was selected. After routine disinfection and draping, a piece of 1.0 × 0.5 cm size tissue was cut with a scalpel, and the wound was sutured and bandaged routinely. Each sample was divided into two pieces, one was fixed and embedded in paraffin, and the other was soaked in dispase solution (1 mg/ml) overnight at 4 °C to separate epidermis and dermis for subsequent experiments. The healthy control group was selected from the normal skin of the patients undergoing plastic surgery. This study was approved by the Ethics Committee of Xijing Hospital of the Fourth Military Medical University (No.ky20173038-1).

### SiRNAs and plasmids

HaCat cells were transfected with siRNAs targeting KLK8 (Ribo biotech, Guangzhou, China) or with plasmid encoding KLK8 (Genecreate biotech, Wuhan, China) using Lipofectamine 3000 according to the manufacturer’s instructions.

### Lentiviral-EZH2-shRNA and Lentiviral-EZH2

Lentiviral vectors were packaged with shRNA targeting EZH2 or full-length Ezh2 gene by Sangon biotech (Shanghai, China). EZH2–shRNA sequences were listed in Supplementary Table 2. HaCat cells were infected with these lentiviral vectors according to the manufacturer’s instructions. The optimal infection concentration of lentivirus is 1 × 10^7^ TU/ml + polybrene. HaCat cells were infected with the lentivirus for 48–72 h, and then were subcultured into 6-well plates or 96-well plates for subsequent experiments.

### Construction of stable EZH2 knockdown HaCat cell line

HaCaT cells infected with lentivirus were transferred into 96-well plate, and the cells were screened by adding different concentrations of puromycin (1.25, 2.5, 5, 10, and 20 μg/ml). After 48 h, the cell survival rate was observed under the light inverted microscope, and the proportion of green fluorescence positive cells was observed under the fluorescence microscope. The optimal concentration of puromycin was 5 μg/ml. HaCaT cells infected with lentivirus were subcultured and inoculated into T25 cell culture bottle. The HaCaT cell line stably knockdown of EZH2 was obtained by three passages screening with 5 μg/ml puromycin.

### Microarray analysis

Three stable EZH2 knockdown HaCat cell lines and three control HaCat cell lines were used for microarray analysis. Total RNA was extracted using TRIzol reagent (Thermo Scientific). Microarrays were performed using Affymetrix GeneChip PrimeView™ Human Gene Expression Array chips (Affymetrix, Santa Clara, CA). The resulting data were analyzed by CapitalBio Technology Company (Beijing, China). A 1.3-fold change cut-off (increased to more than 1.3 times or decreased to less than 0.76 times of the control group) was set to screen differentially expressed genes^36,37^. Accession code for microarray data: 10.5061/dryad.z612jm69b.

### EdU proliferation assay

HaCat cells infected with lentiviral EZH2-shRNA or lentiviral-EZH2 or control lentivirals were subcultured into 96-well plates. Each group had five replicate wells. The next day, siRNA or overexpression plasmid was transfected into cells using liposomes (Thermo Scientific). Twelve hours later, the culture medium was changed to add mixed cytokines, and the culture was continued for 24 h before EdU proliferation assay. EdU proliferation assay was performed by using Cell-light EdU Apollo567 in vitro kit (Ribo biotech) according to the manufacturer’s instructions. After treatment of different groups, cells were incubated with 50 μM of EdU for 2 h at 37 °C.Then the cells were fixed with 4% paraformaldehyde and stained with Apollo staining solution. Images were obtained under a fluorescence microscope (Olympus, Tokyo, Japan). Three different fields of view for each well was taken. Image-Pro Plus 6.0 software was used to count manually the positive cells in the images. Three researchers counted the cells (including bright and less bright cells) separately, and the average of their results was taken as the final results.

### Cell counting kit-8

HaCat cells infected with lentiviral EZH2–shRNA or lentiviral-EZH2 or control lentivirals were subcultured into four 96-well plates for four different observation time point. Each group had five replicate wells. Cells adhered to the wall after 6 h, this time was recorded as observation point 0 h. The next day, siRNA or overexpression plasmid was transfected into cells using liposomes (Thermo Scientific). Twelve hours later, the culture medium was changed to add mixed cytokines, and the culture was continued for 24, 48, and 72 h in different plates. Then cells were incubated with the dilution of Cell Counting Kit-8 (7seapharmtech, Shanghai, China) for 2 h at 37 °C.The absorbance at 450 nm was determined by a microplate reader (Thermo Scientific).

### Animal experiments

Female BALB/c mice aged 6–8 weeks were obtained from the Department of Laboratory Animal Medicine of the Fourth Military Medical University. Mice were randomly assigned to three groups. GSK126 dissolved in absolute ethanol (1.5 mg/ml) was added to the emulsion matrix to prepare a topical drug at a final concentration of 1 mg/ml. The same ingredients without GSK126 was prepared as control (Vehicle). Shaved mouse dorsal skin was topically treated daily with imiquimod (Aldara, INova, Chatswood, Australia) at 8:00 am to induce a psoriatic mouse model^38^ and with GSK126 or Vehicle respectively at 16:00 pm for seven days. On day 8, skin specimens were obtained for analysis. Three independent animal experiments were performed, each with three mice per group. The investigators were not blinded to the group allocation during the experiment and when assessing the outcome. This work was approved by the Experimental Animal Ethics Committee of the Fourth Military Medical University.

### H&E staining

Tissue was soaked in 10% formaldehyde for 48 h at 4 °C. The paraffin section was done according to the usual procedure, and the thickness of the slice was 10 μm. Gradient alcohol was employed to dewax of sections before dyeing. The sections were stained with hematoxylin solution and eosin staining solution successively, dehydrated by gradient alcohol, and then putted in xylene for transparency. Then the sections were sealed with neutral resin. Images were acquired under a Aperio digital pathology slide scanner (Leica, Heerbrugg, Switzerland). Nanozoomer Digital Pathology (NDP) Image software was used to measure epidermal thickness. Five points in each section were measured, and the average of these points was taken as the final results of each sample.

### Immunofluorescence and immunohistochemical staining

The steps before dewaxing (including dewaxing) were the same as H&E staining. Sections were blocked with 1% BSA in PBS and then incubated with primary antibodies. After washing with PBS, sections were incubated with fluorescent secondary antibodies (for immunofluorescence staining) or horseradish peroxidase labeled secondary antibody (for immunohistochemical staining). DAB color development was employed for immunohistochemical staining. Images were acquired under a Aperio digital pathology slide scanner (Leica, Heerbrugg, Switzerland) or FV1000 confocal microscope (Olympus). Image J software was used for immunofluorescence quantification. After the epidermal area of the whole image was circled, the average fluorescence intensity was measured. Image-Pro Plus 6.0 software was used to count manually the Ki67 or PCNA positive cells in the images. Three researchers counted the cells (including bright and less bright cells) separately, and the average of their results was taken as the final results.

### Western blotting

Protein was extracted with RIPA from tissue of cultured cells and boiled with loading buffer for 10 min after BCA protein quantification. Sample was transferred to PVDF membrane after electrophoresis. Then, PVDF membrane was blocked with 1% BSA in PBS followed by incubating with primary antibodies. After washing with PBST, the membrane was incubated with horseradish peroxidase labeled secondary antibody. At last, the membrane was chemiluminescence using the chemiluminescent reagents (Millipore Corporation, Billerica, MA) and image-forming system Bio-Rad ChemiDoc XRS+ (Bio-Rad, Hercules, Calif).

### Real-time PCR

Total RNA was extracted by using the TRIzol reagent (Thermo Scientific) according to the manufacturer’s instructions. cDNA was prepared by using a reverse transcription (RT) system (Takara, Ohtsu, Japan). Quantitative Real-Time PCR was performed in triplicates by using a kit (SYBR Premix EX Taq; Takara) and the iQ5 PCR Detection System (Bio-Rad, Hercules, Calif), with β-actin as an internal control. Primer sequences are listed in Supplementary Table 3.

### Statistical analysis

All data in our article are obtained from at least three independent experiments. Results were expressed as means ± standard deviation (SD). Statistical analysis was performed with the GraphPad Prism software. Comparisons between two groups were undertaken using unpaired Student’s *t*-test. Comparisons of multiple groups were determined using one-way ANOVA followed by Newman–Keuls Multiple Comparison Test. The variance was similar between the groups that are being statistically compared (*P* > 0.05). *P* < 0.05 was considered statistically significant.

## Supplementary information

Supplementary Figure Legends

Supplementary figure 1

Supplementary figure 2

Supplementary figure 3

Supplementary figure 4

Supplementary figure 5
