# Supplementary material for: EZH2-dependent epigenetic modulation of histone H3 lysine-27 contributes to psoriasis by promoting keratinocyte proliferation
Source: Cell Death Dis. 2020 Oct 3;11(10):826. doi: 10.1038/s41419-020-03028-1 (PMC7532974; doi:10.1038/s41419-020-03028-1)
Supplement: Supplementary file 1 — Supplementary Figure Legends [file 41419_2020_3028_MOESM1_ESM.doc]

**Supplementary Figure Legends**

**Supplementary figure 1.** The change of ten selected genes was validated by Real-Time PCR. **P*＜0.05; ***P*＜0.01; ****P*＜0.001.

**Supplementary figure 2. The efficiency of KLK8 siRNA and KLK8 overexpression plasmid.** (A) HaCat cells were transfected with siRNA for KLK8 (KLK8-siRNA) or control siRNA, and treated with mixed cytokines. Protein levels of KLK8, EZH2 and H3K27me3 were detected by Western Blotting. (B) Plasmid for overexpression of KLK8 (Plasmid-KLK8) or control plasmid (Plasmid-Control) was transfected in HaCaT cells. Protein levels of KLK8, EZH2 and H3K27me3 were detected by Western Blotting.

**Supplementary figure 3. GSK126 restrained cell proliferation induced by mixed cytokines.** (A) After treatment with GSK126 and mixed cytokines for 24h，H3K27me3 levels in HaCat cells were detected by Western blot. (B) Proliferation of cells was analyzed by EdU assay. Scale bar＝100 μm. * *P*＜0.05; *** *P*＜0.001.

**Supplementary figure 4. No significant difference was found in EZH1 between psoriatic lesions and normal skin.** EZH1 mRNA levels in psoriatic lesions and normal skin were detected by Real-Time PCR.

**Supplementary figure 5. Down-regulation of H3K27me3 levels in PBMC of psoriasis patients.** (A) H3K27me3 positive cells in CD3 or CD19 positive cells in PBMC of patients with psoriasis were detected by flow cytometry. (B) The H3K27me3^+^ CD3^+^ T lymphocytes in PBMC of patients with psoriasis was significantly lower than that of normal people, and was negatively correlated with PASI score (C).

**Supplementary table 1. Patients statistics**

| **patient ID** | **sex** | **age** | **Diagnosis** | **PASI score** | **Sample location** |
| --- | --- | --- | --- | --- | --- |
| 99664966 | Male | 23 | Psoriasis vulgaris | 18.7 | Upper back |
| 99679327 | Male | 57 | Psoriasis vulgaris, Hypertension | 28.0 | Abdomen |
| 99532274 | Male | 28 | Psoriasis vulgaris | 29.3 | Mid back |
| 97770147 | Male | 24 | Psoriasis vulgaris | 36.8 | Abdomen |
| 99640198 | Female | 47 | Psoriasis vulgaris | 12.9 | Upper back |
| 99620109 | Female | 29 | Psoriasis vulgaris | 19.9 | Mid back |

**Supplementary table 2. EZH2-shRNA sequences**

| Name | Sequences |
| --- | --- |
| Control-shRNA |  |
| Top strand | GATCCGTTCTCCGAACGTGTCACGTAATTCAAGAGATTACGTGACACGTTCGGAGAATTTTTTC |
| Bottom strand | AATTCAAAAAATTCTCCGAACGTGTCACGTAATCTCTTGAATTACGTGACACGTTCGGAGAACG |
| EZH2-shRNA1 |  |
| Top strand | GATCCGCAAATTCTCGGTGTCAAATTCAAGAGATTTGACACCGAGAATTTGCTTTTTTC |
| Bottom strand | AATTGAAAAAAGCAAATTCTCGGTGTCAAATCTCTTGAATTTGACACCGAGAATTTGCG |
| EZH2-shRNA2 |  |
| Top strand | GATCCGGATGGTACTTTCATTGAATTCAAGAGATTCAATGAAAGTACCATCCTTTTTTC |
| Bottom strand | AATTGAAAAAAGGATGGTACTTTCATTGAATCTCTTGAATTCAATGAAAGTACCATCCG |
| EZH2-shRNA3 |  |
| Top strand | GATCCGTCGGTAAATCCAAACTGCTATGCAATTCAAGAGATTGCATAGCAGTTTGGATTTACCGATTTTTTC |
| Bottom strand | AATTGAAAAAATCGGTAAATCCAAACTGCTATGCAATCTCTTGAATTGCATAGCAGTTTGGATTTACCGACG |

**Supplementary table 3. Primer sequences for Real-Time PCR**

| Gene name | Primer sequences(5'→3') | |
| --- | --- | --- |
| EZH2 | F: | TTGTGACAGTTCGTGCCCTT |
|  | R: | AGCGGCTCCACAAGTAAGAC |
| CCND1 | F: | CAGAAGTGCGAGGAGGAGGT |
|  | R: | TAGAGGCCACGAACATGCAA |
| CDK6 | F: | TCAGCTTCTCCGAGGTCTGG |
|  | R: | AAGGCCGAAGTCAGCGAGT |
| FGFR2 | F: | CCGAATGAAGAACACGACCA |
|  | R: | TCATGGAGGAGCTGGACTCA |
| ESM1 | F: | GCATGGATGGCATGAAGTGT |
|  | R: | GCAGTTGCAGGTCTCTCTGC |
| TGM2 | F: | CAACACCGCTGAGGAGTACG |
|  | R: | AGGCTCCAGGTTGAGGTTGA |
| KLK8 | F: | GAGAACTTGCCGTGTGTCCA |
|  | R: | CAGCACCTTGTCCTCCTGTG |
| RBPJ | F: | CAGTCCGAGATGGCTACATCC |
|  | R: | CATCCAATAATGCGGTCTGC |
| DUSP6 | F: | CTGCATTGCGAGACCAATCT |
|  | R: | CGATGTCCGAGGAAGAGTCA |
| KRT17 | F: | GGTGCAGAGTGGCAAGAGTG |
|  | R: | GTTCTCTGTCTCCGCCAGGT |
| KLK6 | F: | TGACACCATCCAGTGTGCAT |
|  | R: | TCGGAGGTGGTCTCCACATA |
| ISG15 | F: | CTGAGCATCCTGGTGAGGAA |
|  | R: | CCTCGAAGGTCAGCCAGAAC |
| EED | F: | ACGTGGATGCTGATGCTGAT |
|  | R: | GCTACAGCCAGCAGAGGATG |
| EZH1 | F: | CAACTTCTGTTCAATAGCCAGG |
|  | R: | TTTTCTTCTTCTGTGAGGGGTT |
| β-actin | F: | TGGCACCCAGCACAATGAA |
|  | R: | CTAAGTCATAGTCCGCCTAGAAGCA |
