# Supplementary figures and images for: EZH2-dependent epigenetic modulation of histone H3 lysine-27 contributes to psoriasis by promoting keratinocyte proliferation
Source: Cell Death Dis. 2020 Oct 3;11(10):826. doi: 10.1038/s41419-020-03028-1 (PMC7532974; doi:10.1038/s41419-020-03028-1)

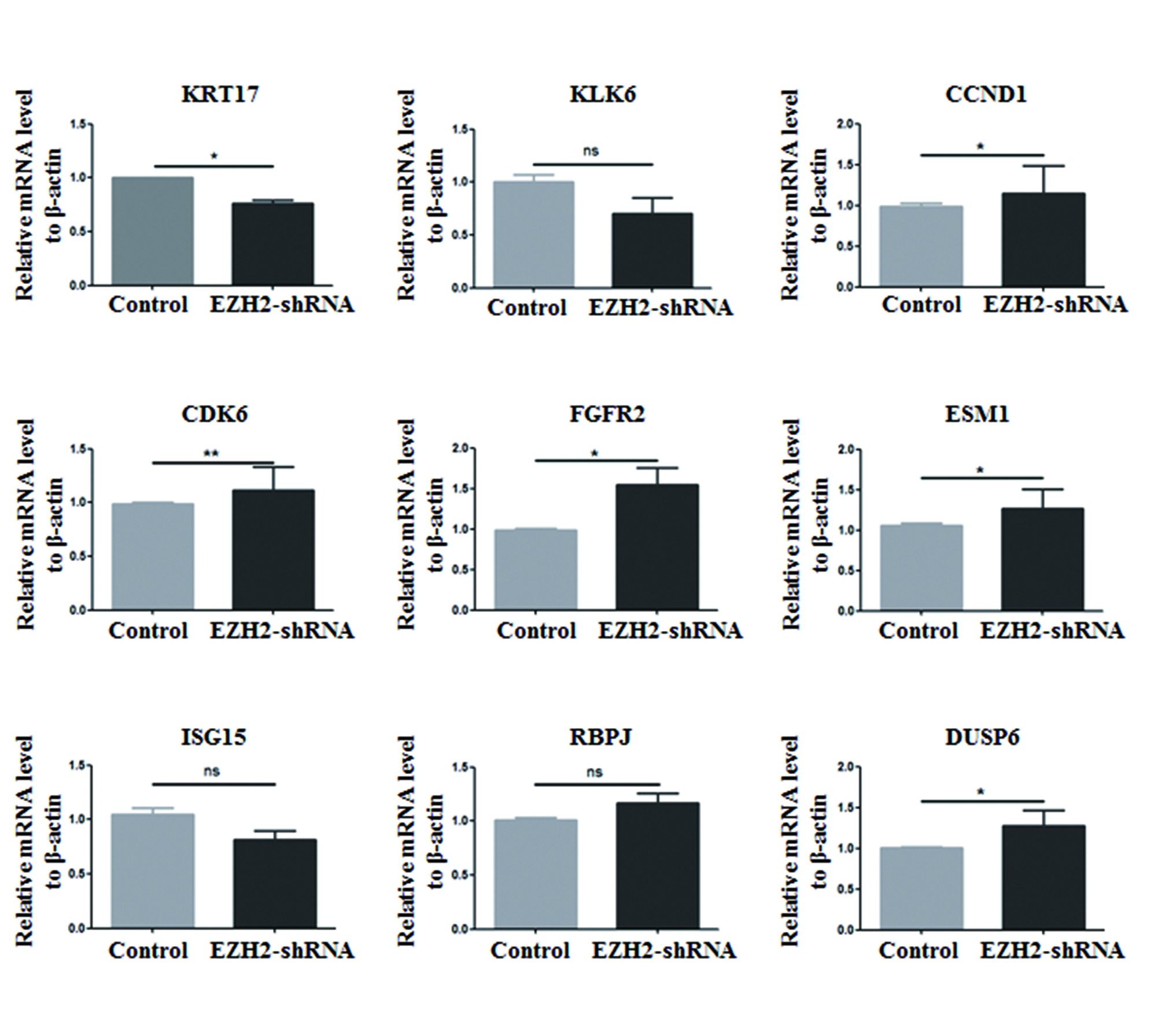

Supplement: Supplementary file 2 — Supplementary figure 1 [file 41419_2020_3028_MOESM2_ESM.tif]

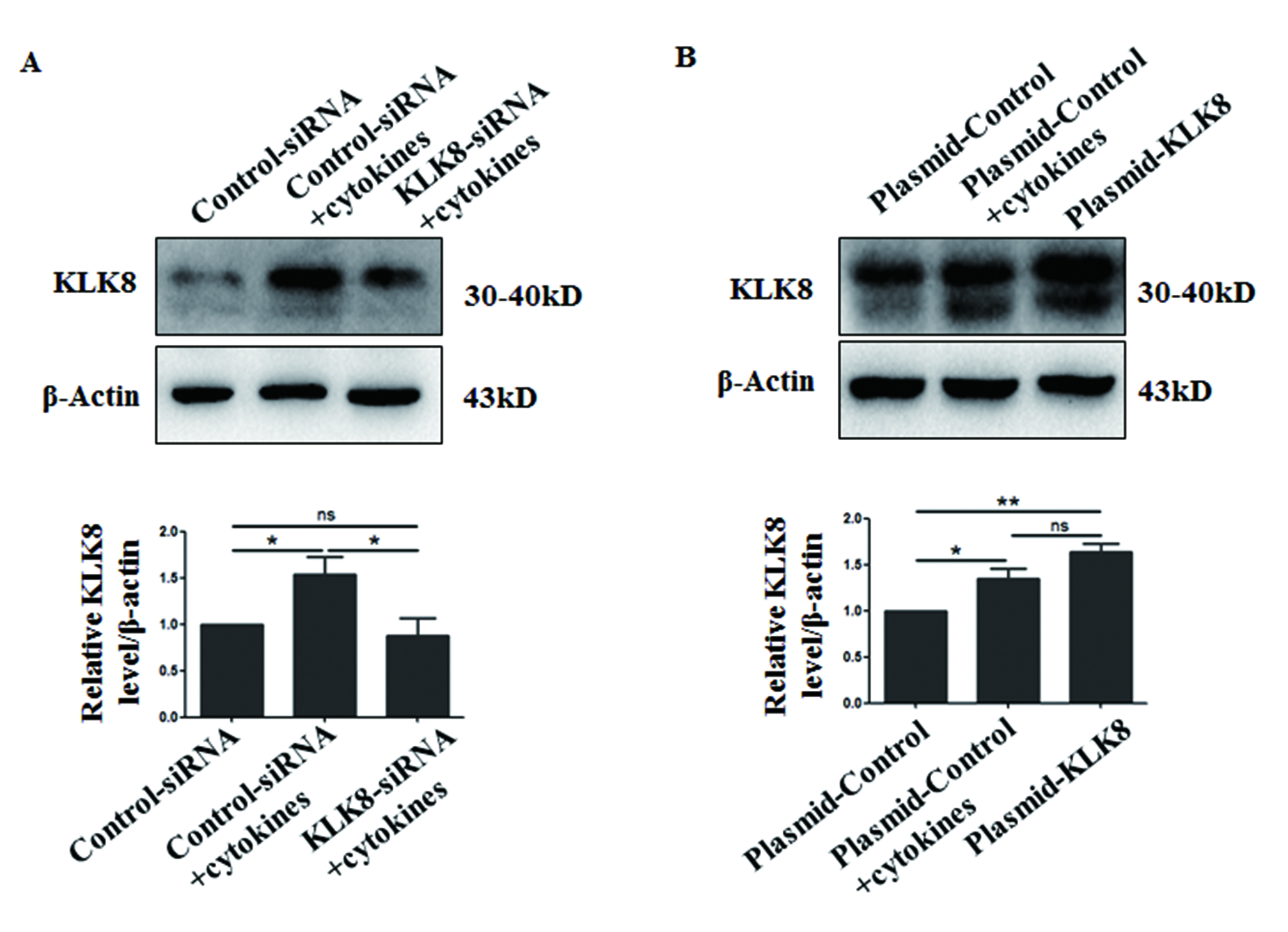

Supplement: Supplementary file 3 — Supplementary figure 2 [file 41419_2020_3028_MOESM3_ESM.tif]

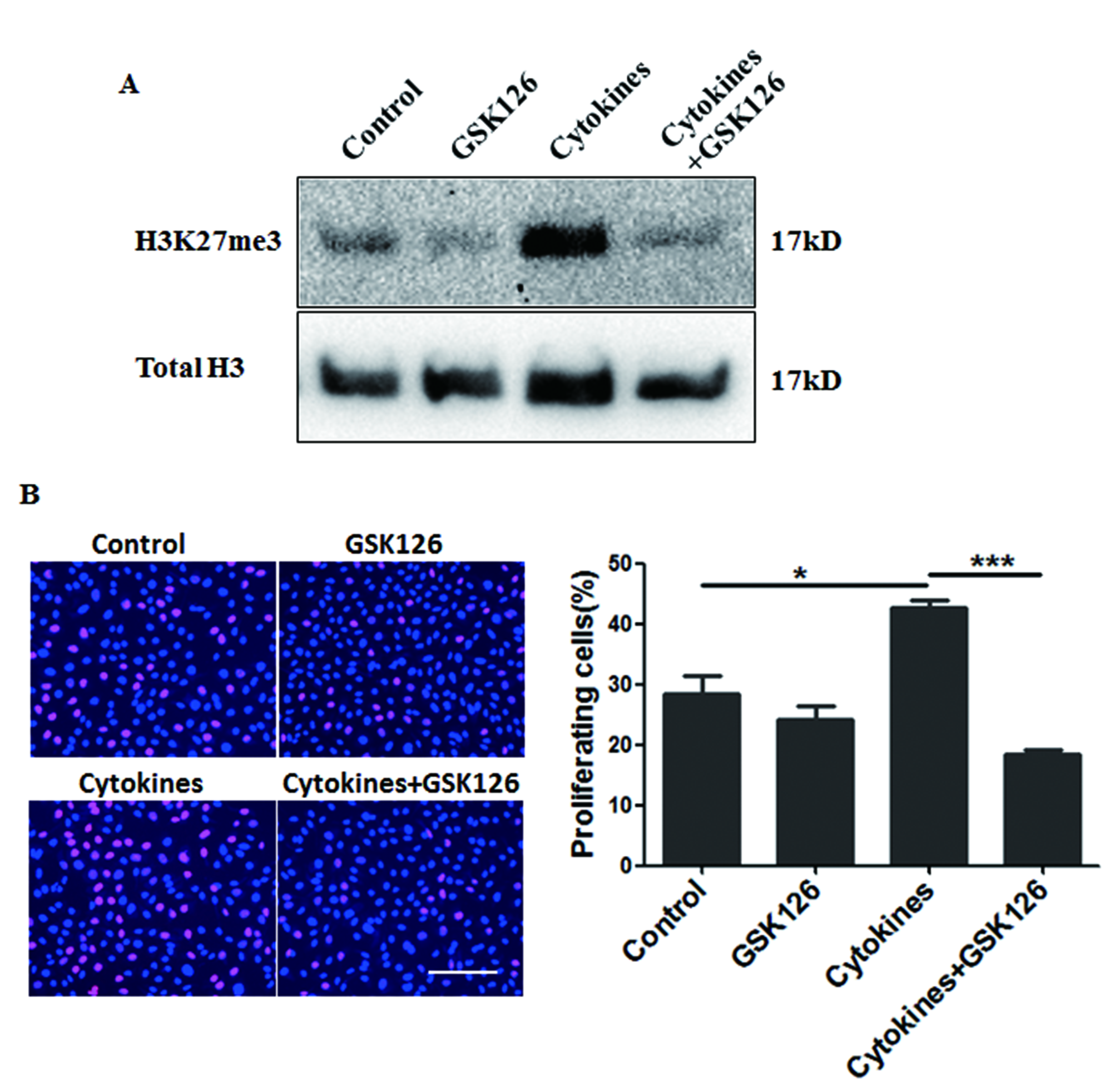

Supplement: Supplementary file 4 — Supplementary figure 3 [file 41419_2020_3028_MOESM4_ESM.tif]

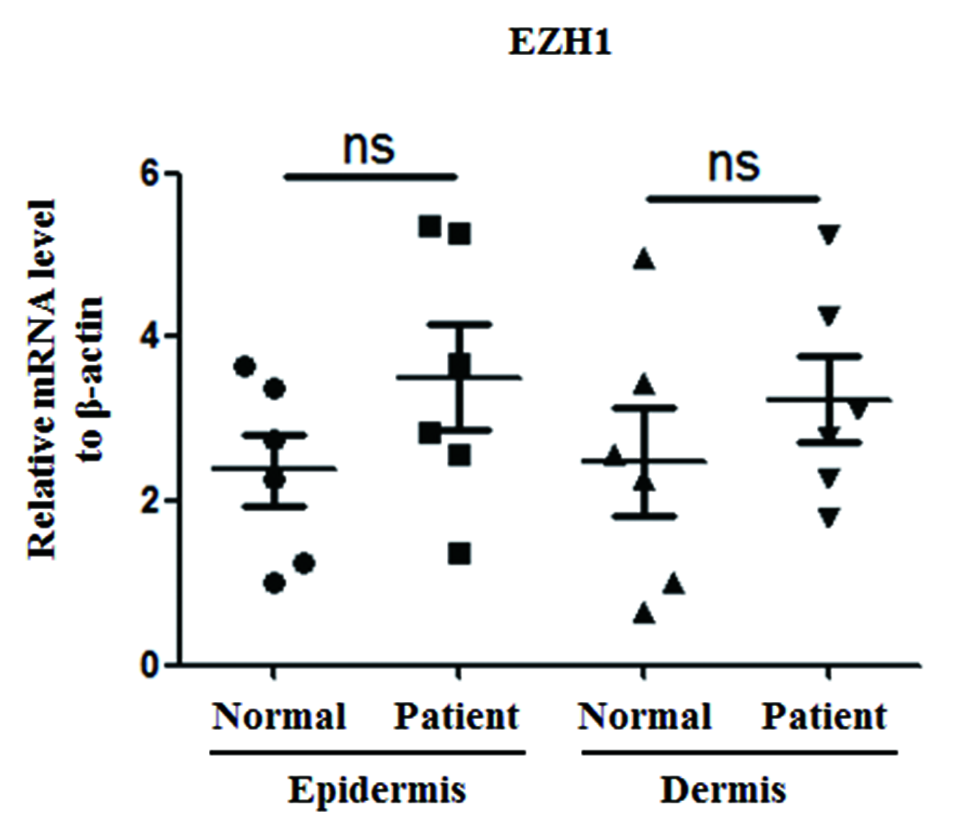

Supplement: Supplementary file 5 — Supplementary figure 4 [file 41419_2020_3028_MOESM5_ESM.tif]

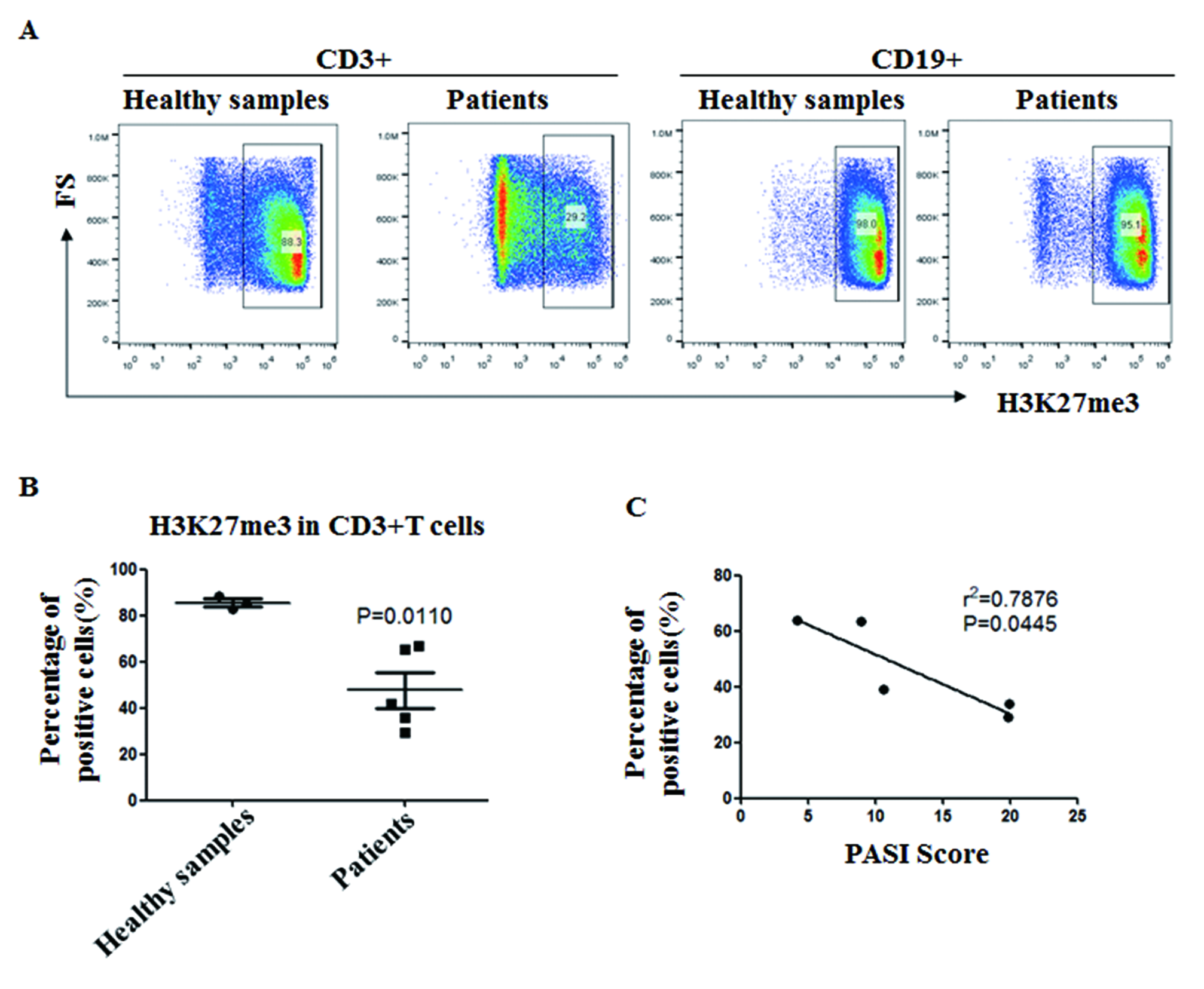

Supplement: Supplementary file 6 — Supplementary figure 5 [file 41419_2020_3028_MOESM6_ESM.tif]
